# Supplementary material for: The interactome of intact mitochondria by cross-linking mass spectrometry provides evidence for coexisting respiratory supercomplexes
Source: Mol Cell Proteomics. 2017 Dec 8;17(2):216–32. doi: 10.1074/mcp.RA117.000470 (PMC5795388; doi:10.1074/mcp.RA117.000470)
Supplement: Supplemental Data [file supp_RA117.000470_133922_0_supp_23978_fzffwf.zip › spectra_annotation/mito_DR_spectra_annotation/202-1-3-1-16-1.pdf]

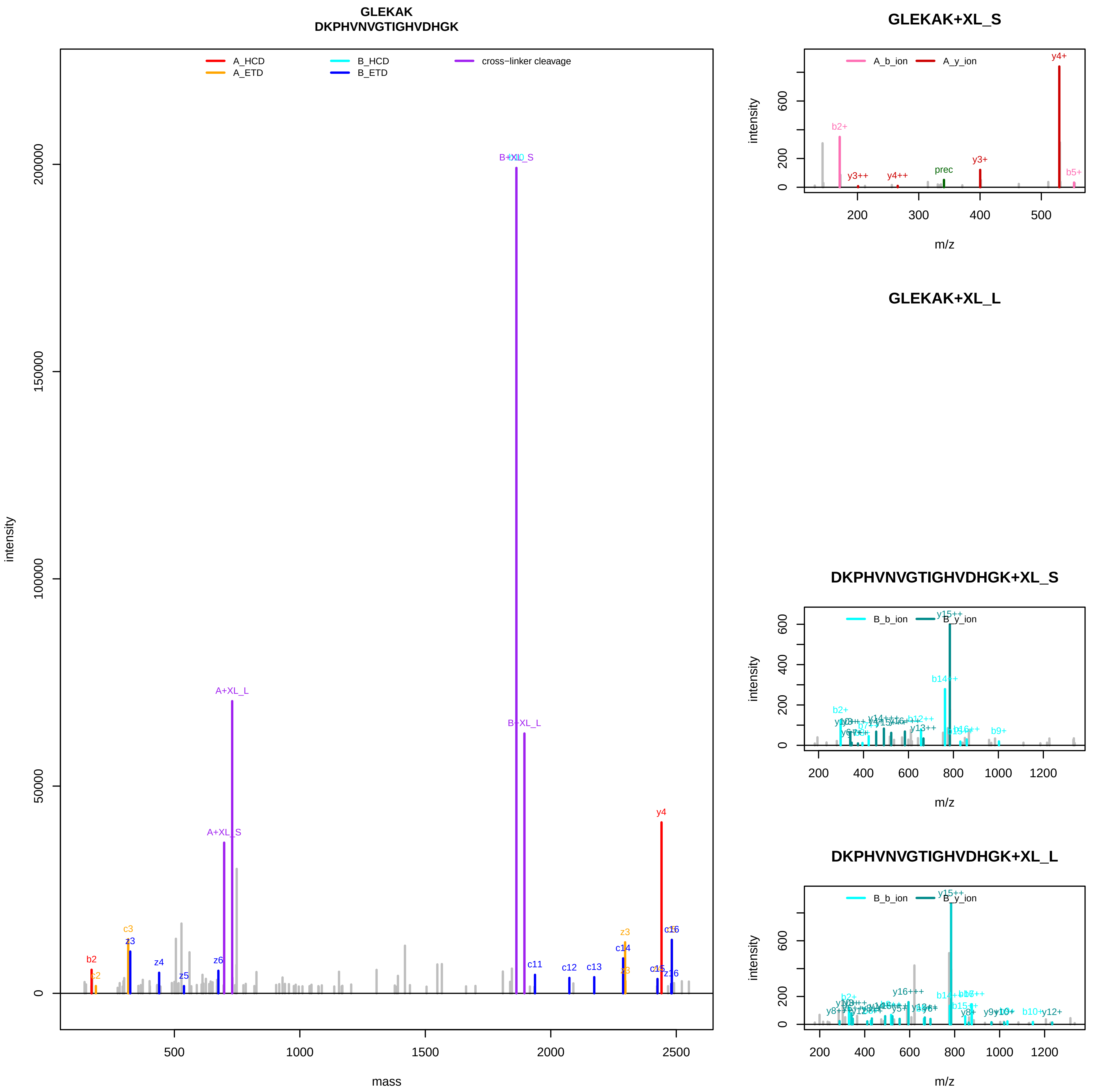
GLEKAK
DKPHVNVGTIGHVDHGK
A_HCD
A_ETD
B_HCD
B_ETD
cross−linker cleavage
intensity
mass
0
50000
100000
150000
200000
500
1000
1500
2000
2500
B+XL_S
A+XL_L
B+XL_L
A+XL_S
y4
b2
c2
c3
z3
z4
z5
z6
c11
c12
c13
c14
c15
c16
z16
GLEKAK+XL_S
A_b_ion
A_y_ion
y4+
b2+
y3++
y4++
prec
y3+
b5+
m/z
GLEKAK+XL_L
DKPHVNVGTIGHVDHGK+XL_S
B_b_ion
B_y_ion
y15++
b14++
b2+
b9+
y13++
DKPHVNVGTIGHVDHGK+XL_L
y15++
y16+++
b2+
y8+
b10+
y12+
